# Distinct 24-hour movement behaviour profiles and their associations with negative affect among Chinese university students

**DOI:** 10.1186/s40359-026-04889-5

**Published:** 2026-05-29

**Authors:** Yi Chen, Xinshuai Guo, Wenrui Zhao

**Affiliations:** College of Physical Education and Health Sciences, Zhejiang Normal University, Jinhua, 321004 China

**Keywords:** 24-hour movement behaviours, Latent profile analysis, Sedentary behaviour, Physical activity, Depression, Anxiety, Stress

## Abstract

**Background:**

University students experience a high prevalence of depression, anxiety and stress, while simultaneously undergoing substantial changes in daily movement behaviours. From a 24-hour movement behaviour perspective, sleep, sedentary behaviour and physical activity are interdependent components of a fixed time budget, yet little is known about how distinct combinations of these behaviours cluster among Chinese university students and how such patterns relate to negative affect.

**Methods:**

A cross-sectional online survey was conducted from October 2024 to April 2025 using stratified cluster and simple random sampling across universities in Shandong, Zhejiang and Tianjin, yielding 1,056 valid responses. The 24-Hour Movement Behaviour Questionnaire (24HMBQ) assessed sleep, sedentary behaviour and physical activity. Negative affect was measured using the Depression Anxiety Stress Scales-21 (DASS-21). Latent Profile Analysis (LPA) was used to identify behavioural profiles, and the R3STEP and Bolck–Croon–Hagenaars (BCH) approaches were used to examine demographic predictors and differences in negative affect.

**Results:**

A two-profile solution was retained as the primary representation of 24-hour movement behaviours among Chinese university students, comprising a lower-activity profile (86.0%) and a higher-activity profile (14.0%). Compared with the lower-activity profile, the higher-activity profile showed substantially higher levels of vigorous-, moderate-, and light-intensity physical activity, lower screen-based sedentary behaviour, and slightly higher study-related sedentary time. Gender and academic year significantly predicted profile membership, with female students being less likely and junior students more likely to belong to the higher-activity profile. BCH analyses further showed that students in the lower-activity profile reported significantly higher levels of depression, anxiety, and stress than those in the higher-activity profile.

**Conclusions:**

Chinese university students showed heterogeneity in 24-hour movement behaviours, but the most robust distinction was between lower-activity and higher-activity patterns. Lower-activity patterns were associated with less favourable emotional outcomes. These findings underscore the importance of an integrated 24-hour movement perspective and suggest that interventions aimed at reducing screen-based sedentary behaviour and increasing physical activity may help promote emotional wellbeing in university students.

**Supplementary Information:**

The online version contains supplementary material available at 10.1186/s40359-026-04889-5.

## Introduction

 The mental health of university students is fundamental to their personal development, academic achievement and future social adaptation. Yet a recent meta-analysis of university populations worldwide indicates a worrying global prevalence of negative emotional states. Symptoms such as anxiety, depression and stress occur at notably high rates, with comorbid anxiety and depression affecting 33.6% and 39.0% of students respectively, and a substantial proportion simultaneously experiencing elevated psychological stress [1]. In China, the combined pressures of intense academic expectations and fierce social competition further compound students’ psychological burden, with evidence suggesting that 35–40% of Chinese university students have exhibited a marked rise in depressive or anxious symptoms [2, 3]. These negative emotional experiences undermine academic performance, social relationships and subjective wellbeing, and constitute major precursors of academic burnout, emotional disorders and even increased suicide risk [4–7]. Identifying modifiable behavioural risk factors and understanding their relationships with negative affect is therefore essential for informing effective preventive and intervention strategies to support student mental health.

Alongside the rising prevalence of mental-health concerns, university students have also experienced marked shifts in their everyday behavioural patterns. The recently proposed 24-hour movement behaviour paradigm conceptualises sleep, sedentary behaviour and physical activity as interdependent components of a fixed time budget, wherein increases in one necessarily displace time spent on the others [8]. For university students, this life stage is characterised by distinctive behaviour patterns: demanding course schedules and examination pressure often lead to prolonged study-related sitting; the pervasive use of digital technologies results in extensive daily screen time on mobile phones and computers; and irregular sleep routines, late-night screen use and shared dormitory environments contribute to insufficient sleep, poorer sleep quality and disrupted circadian rhythms. Evidence suggests that a considerable proportion of Chinese university students do not adhere to a balanced 24-hour behavioural pattern, instead devoting substantial time to study-related sitting, engaging extensively in screen-based sedentary behaviour, and participating insufficiently in moderate-to-vigorous physical activity. These patterns are frequently accompanied by delayed bedtimes, shortened sleep duration and irregular routines [9, 10]. Such tendencies point towards the possibility that students’ behavioural patterns are shaped by the distinctive educational and sociocultural context of Chinese higher education. On the one hand, an academic culture that places high value on performance and examination outcomes produces substantial study burdens, contributing to lengthy periods of study-related sitting. On the other, the widespread use of digital teaching tools, online social platforms and entertainment media substantially increases screen-based sedentary time, creating a dual pressure of “academic sitting” and “screen sitting” that may be particularly salient among Chinese students [11]. Meanwhile, intensive course timetables, limited access to campus sports facilities and varying levels of institutional emphasis on physical exercise collectively restrict opportunities for engagement in physical activity, resulting in generally low activity levels. Cultural factors—including the stigmatisation of mental illness and insufficient mental-health literacy—further contribute to students’ reluctance to seek professional psychological support. Many students prefer to conceal or self-manage emotional difficulties rather than pursue formal counselling. Existing research shows that self-stigma significantly weakens students’ attitudes towards help-seeking, and that low mental-health literacy reinforces stigma and discrimination, thereby further discouraging formal support-seeking [12, 13]. Against this backdrop, clarifying the associations between distinct 24-hour movement behaviour profiles and negative affect is of substantial importance. Theoretically, such work can enrich cross-cultural evidence on the links between movement behaviours and mental health and deepen understanding of the mechanisms through which behavioural patterns shape psychological outcomes. Practically, it can provide Chinese universities with empirically grounded, profile-specific strategies for mental-health promotion and targeted interventions, thereby offering meaningful guidance for supporting student wellbeing. 

### 24-hour movement behaviours and negative affect

Existing research demonstrates that the various components of 24-hour movement behaviour exert differential effects on negative affect. Systematic reviews and longitudinal studies consistently show that university students who obtain sufficient and high-quality sleep exhibit substantially lower risks of depression, anxiety and psychological distress, whereas insufficient sleep, poor sleep quality and circadian rhythm disruption are strongly associated with heightened risks of these conditions [14]. Sedentary behaviour—particularly prolonged screen time related to entertainment or social media—has been linked to increased social comparison, reduced offline social support and poorer interpersonal functioning, and is now recognised as a major risk factor for anxiety and depression among young people [15, 16]. In contrast, regular physical activity exerts substantial protective effects against anxiety and depression through mechanisms such as enhanced neural plasticity, reduced systemic inflammation and improved self-efficacy [17, 18]. It is important to note, however, that these behaviours rarely occur in isolation; increases in one inevitably reduce time spent on others. For instance, excessive screen use is typically accompanied by reduced sleep and lower levels of physical activity. Reflecting this integrative perspective, Canada released the *Canadian 24-Hour Movement Guidelines for Adults Aged 18–64 Years* in 2020, which provide specific recommendations for sleep, sedentary behaviour and physical activity. The guidelines advocate shifting attention away from isolated behaviours toward the full composition of the 24-hour cycle, recommending 7–9 h of quality sleep per day, no more than 8 h of sedentary time, and at least 150 min of moderate-to-vigorous physical activity per week [19]. Accordingly, examining the combined patterns formed by sleep, distinct types of sedentary behaviour and multiple intensities of physical activity—rather than studying each behaviour in isolation—offers a more scientifically robust and conceptually meaningful approach to understanding their associations with negative affect.

### Person-centred approaches to movement behaviour research

Most prior studies have used variable-centred approaches to examine single behavioural indicators, which cannot capture the combined patterns of multiple daily behaviours. Latent Profile Analysis (LPA), a person-centred method, identifies heterogeneous subgroups based on behavioural compositions and thus offers a more holistic perspective [20]. Evidence shows that profiles marked by high sedentary time and low moderate-to-vigorous activity are linked to higher depressive symptoms, whereas more active and less sedentary profiles are associated with better mental health [21, 22]. Applying LPA to university students’ 24-hour movement behaviours therefore enables the identification of meaningful behavioural patterns and high-risk combinations relevant to psychological wellbeing.

### The present study

The present study pursued two primary objectives. First, we aimed to identify latent profiles of 24-hour movement behaviour among Chinese university students using Latent Profile Analysis, based on seven behavioural dimensions: sleep, study-related sitting, screen-based sitting, other sedentary behaviour, and light-, moderate- and vigorous-intensity physical activity. Second, we examined the associations between the identified profiles and students’ negative emotional outcomes, including anxiety, depression and stress. By integrating behavioural typologies with multidimensional assessments of negative affect, this study seeks to illuminate more nuanced links between students’ daily behavioural patterns and psychological risks. In doing so, it provides an empirical foundation for the development of targeted mental-health promotion and precision intervention strategies within higher-education settings.

Based on prior research on 24-hour movement behaviours among adolescents and young adults, we expected to identify approximately three to four profiles, including a sedentary-dominant group (characterised by high sedentary time and low physical activity), a high-activity group (characterised by higher levels of moderate-to-vigorous physical activity), and one or more intermediate profiles (e.g., a lightly active group).

In terms of behavioural indicators, we anticipated that sedentary behaviour and moderate-to-vigorous physical activity would serve as the primary distinguishing features across profiles, while sleep and light-intensity physical activity might play a secondary role in differentiating behavioural patterns. Regarding negative affect, we hypothesised that students in the sedentary-dominant profile would report the highest levels of depression, anxiety, and stress, whereas those in the high-activity profile would exhibit the lowest levels of negative affect.

Although these hypotheses were informed by prior literature, the present study remained exploratory in nature. Latent profile analysis was used as a person-centred technique to identify the optimal number and structure of behavioural profiles without imposing a priori class solutions. The study hypotheses and analytic plans were not preregistered.

## Methods

### Participants

Data were collected between October 2024 and April 2025 using a combination of simple random sampling and stratified cluster sampling. The participants were full-time university students drawn from multiple higher-education institutions across Shandong, Zhejiang and Tianjin. Detailed demographic characteristics are presented in Table 1. All participants completed an online questionnaire by scanning a QR code using a mobile phone or smart device, via Wenjuanxing, a widely used online survey platform in China. A total of 1,148 questionnaires were collected; after excluding invalid responses with abnormal completion times or logical inconsistencies, 1,056 valid questionnaires remained, yielding a valid response rate of 92.0%.

**Table 1:** Sample characteristics of this study (*n* = 1056)

Variable	*n*	Percentage(%)
Gender		
Male	436	41.29
Female	620	58.71
Grade		
Freshman	530	50.19
Sophomore	301	28.50
Junior	72	6.82
Senior	79	7.48
Graduate Student	74	7.01
Residential area		
Urban	395	37.41
Rural	661	62.59
Only Child		
Yes	288	27.27
No	768	72.73
Family Economic Status		
Relatively Poor	221	20.93
Average	775	73.39
Relatively Wealthy	60	5.68

## Measurement

### 24-hour movement behaviour

24-hour movement behaviour patterns over the past week were assessed using the Chinese College Student 24-Hour Movement Behaviour Questionnaire (24HMBQ) developed by Zheng et al. [23]. The questionnaire comprises 26 items across three domains: physical activity, sedentary behaviour and sleep. The sleep section assesses average daily sleep duration over the previous week (e.g., “During the past week, what were your usual bedtime and wake-up time each day?”). The sedentary behaviour section measures average daily time spent sitting for study or work (e.g., attending classes or self-study), screen-based sedentary time (e.g., sitting or lying down while using electronic devices for leisure), and other sedentary activities (e.g., sitting while eating or commuting). The physical activity section assesses time spent in vigorous-, moderate- and light-intensity activities. For each intensity level, the questionnaire records time spent across three contexts: structured exercise, active transportation, and daily activities in dormitory life. The durations reported for these three contexts were summed to obtain the total weekly duration for each intensity level. In accordance with the scoring manual, all behaviours were converted into daily averages using the following formulas: sleep and sedentary time = (weekday duration × 5 + weekend duration × 2) / 7; physical activity = total weekly activity duration / 7. The seven indicators used in the latent profile analysis—sleep duration, study-related sedentary behaviour, screen-based sedentary behaviour, other sedentary behaviour, vigorous physical activity, moderate physical activity, and light physical activity—were all derived from the 24HMBQ and represent the key behavioural dimensions assessed by the instrument. The 24HMBQ has demonstrated strong reliability and validity among Chinese university students. The full Chinese version of the questionnaire is provided in Supplementary Table S1, with its corresponding English translation in Supplementary Table S2.

### Negative affect

Negative emotional states were assessed using the Chinese version of the Depression Anxiety Stress Scales-21 (DASS-21), originally developed by Lovibond and Lovibond and later translated and revised by Gong et al. [24]. The scale uses a four-point Likert response format and comprises 21 items across three subscales—depression, anxiety and stress—each containing seven items scored from 0 (“did not apply to me at all”) to 3 (“applied to me most of the time”). Subscale scores were calculated by summing the item scores and multiplying by two, resulting in a range of 0–42. The total DASS-21 score, obtained by summing the three subscale scores (range 0–126), reflects overall levels of negative affect, with higher scores indicating more severe symptoms [25]. The DASS-21 has been widely validated among Chinese university populations. In the present study, Cronbach’s α for the total scale was 0.926, with α coefficients of 0.906, 0.864 and 0.882 for the depression, anxiety and stress subscales, respectively. Confirmatory factor analysis indicated good model fit and satisfactory convergent and discriminant validity. Detailed psychometric statistics are provided in Supplementary Tables S3–S4.

### Statistical analysis

Prior to the main analyses, data screening and cleaning procedures were conducted. First, to identify careless or inattentive responders, questionnaire completion time was examined. Cases with completion times of less than 5 min were excluded. Second, due to the forced-response design of the online survey platform, there were no missing data for any of the key study variables. Third, outliers were screened using standardised scores and inspection of variable distributions. Statistical assumptions were also evaluated: normality was assessed using skewness and kurtosis values, all of which fell within acceptable ranges (|skewness| < 2 and |kurtosis| < 7). After these screening procedures, the final sample comprised 1,056 valid responses. Data analyses were conducted using SPSS 27.0 and Mplus 9.0. Prior to substantive modelling, SPSS 27.0 was used to assess common method bias, evaluate scale reliability and perform descriptive statistics and correlation analyses to examine the distributions and interrelations of the main variables. Subsequently, LPA was performed in Mplus 9.0 using seven indicators of 24-hour movement behaviour—sleep, study-related sitting, screen-based sitting, other sedentary behaviour, and vigorous-, moderate- and light-intensity physical activity. Models specifying one to five profiles were estimated, and the primary profile solution was selected in accordance with established criteria for mixture modelling [26], including the Akaike Information Criterion (AIC), Bayesian Information Criterion (BIC) [27], adjusted BIC (aBIC), entropy, the Lo–Mendell–Rubin likelihood ratio test (LMR) [28], and the bootstrap likelihood ratio test (BLRT). Lower AIC, BIC and aBIC values indicated superior overall model fit, whereas significant LMR and BLRT p-values suggested improved fit when additional profiles were added. Entropy, ranging from 0 to 1, was used to evaluate the accuracy of profile classification, with higher values reflecting greater precision. A minimum class size of at least 5% of the sample was also considered acceptable [29]. Parsimony and substantive interpretability were also considered when selecting the primary profile solution. Because the original a priori hypothesis anticipated approximately three to four profiles, the three-profile solution was examined in relation to the originally expected structure and is reported in the Supplementary Materials. In addition, a more parsimonious two-profile solution was examined as a non-a-priori alternative model. As described below, this two-profile solution was retained as the main analytical model because it provided a clearer and more interpretable distinction between students with lower versus higher activity levels. After identifying the primary latent profile solution, chi-square tests were used to examine differences in demographic characteristics across profiles, and independent-samples t tests were used to compare behavioural indicators between profiles. Regression analyses were then conducted to examine demographic predictors of profile membership. Finally, to assess differences in the distal outcomes of depression, anxiety and stress across profiles, the Bolck–Croon–Hagenaars (BCH) method was employed in Mplus. As a robust three-step approach, the BCH procedure incorporates classification uncertainty into the model and estimates mean differences in continuous distal variables^1^(i.e., negative affect scores) without altering the established profile structure. By avoiding bias introduced through class re-estimation, the BCH method is regarded as a preferred technique for analysing distal outcomes in latent profile models [30]. All statistical tests were two-tailed with a significance threshold of *p* < 0.05.

## Results

### Common method bias test

Common method bias was examined using Harman’s single-factor test. The unrotated factor analysis extracted three factors, with the first factor accounting for 40.808% of the variance, which is below the commonly accepted threshold of 50%. These results indicate that common method bias is unlikely to pose a serious concern in this study [31].

### Descriptive statistics and correlations

Table 2 presents the descriptive statistics and the Pearson correlation matrix for all study variables. Students averaged 542.29 min of sleep daily (approximately 9.0 h). Sedentary time was dominated by study-related sitting (M = 311.92 min), while physical activity consisted primarily of light-intensity movement. Key correlations revealed that longer sleep was associated with less sedentary behaviour (*r* = -0.19 to -0.21, *ps* < 0.01) and more vigorous activity (*r* = 0.11, *p* < 0.01). Higher engagement in moderate-to-vigorous physical activity was consistently linked to lower levels of depression, anxiety, and stress (*r* = -0.08 to -0.12, *ps* < 0.01). Depression, anxiety, and stress scores were positively intercorrelated (*r* = 0.51 to 0.53, *ps* < 0.01).

**Table 2:** Descriptive statistics and correlation analysis

Variable	1	2	3	4	5	6	7	8	9	10
1 Sleep	1									
2 SB: Learning	− 0.191^**^	1								
3 SB: Screen	− 0.210^**^	− 0.055	1							
4 SB: Other	0.042	− 0.056	− 0.048	1						
5 VPA	0.109^**^	− 0.013	− 0.175^**^	0.046	1					
6 MPA	0.044	0.090^**^	− 0.152^**^	0.015	0.534^**^	1				
7 LPA	− 0.088^**^	− 0.114^**^	− 0.177^**^	0.028	0.141^**^	0.266^**^	1			
8 Depression	0.064^*^	0.070^*^	0.045	0.030	− 0.081^**^	− 0.097^**^	− 0.056	1		
9 Anxiety	0.100^**^	0.054	0.050	− 0.018	− 0.089^**^	− 0.118^**^	− 0.070^*^	0.510^**^	1	
10 Stress	0.055	0.070^*^	0.048	− 0.014	− 0.101^**^	− 0.108^**^	− 0.041	0.526^**^	0.534^**^	1
M	542.29	311.92	234.20	87.29	38.79	37.59	83.34	6.32	6.40	7.49
SD	59.42	118.30	102.50	31.20	36.67	31.67	64.92	8.12	7.39	8.04
Min	317.14	30.00	30.00	25.63	0.00	0.00	6.00	0	0	0
Max	732.86	660.00	557.14	257.14	240.00	222.60	448.20	42	42	42

^*^p < 0.05,^**^p < 0.01. *SB* sedentary behaviour, *VPA* vigorous physical activity, *MPA* moderate physical activity, *LPA* light physical activity

### Latent profile analysis

**Fig. 1:**
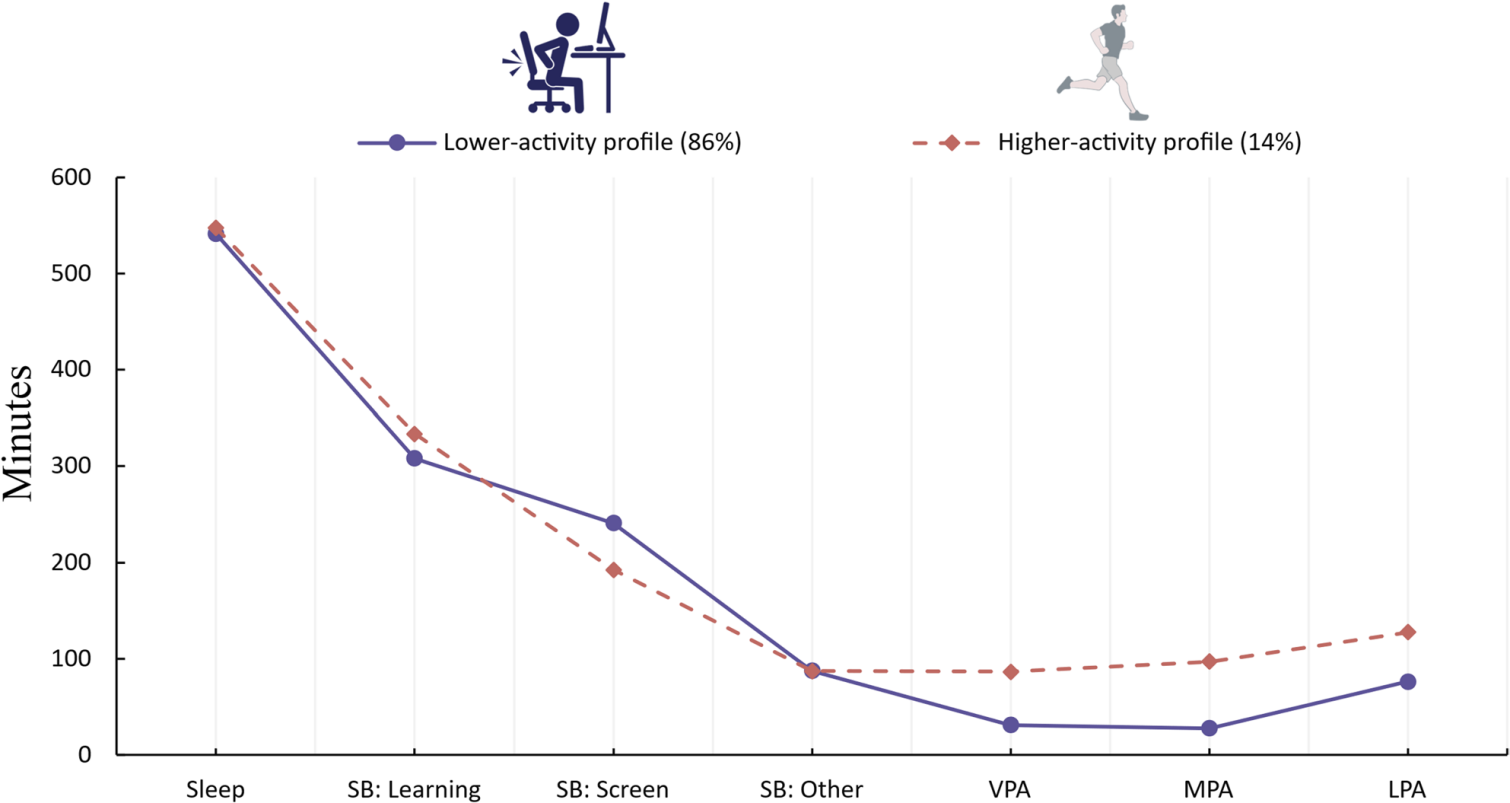
Two-profile model of 24-hour movement behaviours identified in the present study. Note: SB, sedentary behaviour; VPA, vigorous physical activity; MPA, moderate physical activity; LPA, light physical activity.

To identify the underlying patterns of 24-hour movement behaviours among Chinese university students, LPA was conducted using seven behavioural indicators: sleep, study-related sitting, screen-based sitting, other sedentary behaviour, and vigorous-, moderate-, and light-intensity physical activity. Table 3 summarises the model fit indices, entropy values, and class proportions for models specifying one to five profiles. As the number of profiles increased, the AIC, BIC, and aBIC values decreased consistently, indicating progressive improvement in overall fit. The two-profile solution showed the highest entropy (0.911), with both the LMR and BLRT reaching significance (*ps* < 0.001), and yielded acceptable class proportions (86% and 14%). Although the three-profile solution demonstrated lower information criteria than the two-profile solution and significant LMR (*p* = 0.047) and BLRT (*p* < 0.001) values, the two-profile solution provided a more parsimonious and substantively clearer distinction between classes. In addition, the five-profile solution contained a class with only 2% of the sample, raising concerns regarding its practical interpretability and stability. Accordingly, the two-profile solution was retained as the primary model for subsequent analyses, while the three-profile solution was examined as a supplementary analysis and is reported in the Supplementary Materials. The conditional means for each behavioural indicator in the two-profile solution are illustrated in Fig. 1. Profile 1 was labelled the lower-activity profile, and Profile 2 was labelled the higher-activity profile.

**Table 3:** Fit indices for latent profile models with one to five profiles

Model	AIC	BIC	aBIC	Entropy	LMR	BLRT	Group size for each profile(%)
1-profile	80469.59	80539.06	80494.60	-	-	-	-
**2-profile**	**79830.42**	**79939.59**	**79869.71**	**0.911**	***<*** **0.001**	***<*** **0.001**	**0.86/0.14**
3-profile	79594.49	79743.36	79648.08	0.865	0.047	*<* 0.001	0.13/0.73/0.14
4-profile	79427.17	79615.74	79495.04	0.879	0.386	*<* 0.001	0.71/0.13/0.14/0.02
5-profile	79215.72	79443.99	79297.88	0.882	0.008	*<* 0.001	0.12/0.07/0.11/0.68/0.02

Values in bold denote the selected model

*AIC* Akaike Information Criterion, *BIC* Bayesian Information Criterion, *aBIC* adjusted BIC,* LMR * *p* values for Lo-Mendell-Rubin adjusted likelihood ratio test for K vs. K-1 profiles, *BLRT * *p* values for Bootstrapped Likelihood Ratio Test

To further characterise the two latent profiles, the means and standard deviations of the behavioural indicators across profiles are presented in Table 4. Compared with the lower-activity profile, the higher-activity profile reported significantly higher levels of vigorous, moderate, and light physical activity, as well as significantly lower screen-based sedentary behaviour (*ps* < 0.001). The higher-activity profile also showed slightly higher learning-related sedentary behaviour (*p* = 0.011), whereas no significant between-profile differences were observed for sleep or other sedentary behaviour. Among all behavioural indicators, the largest between-profile differences were observed for moderate physical activity, vigorous physical activity, and light physical activity (*d* = 0.808 to 3.579). These findings indicate that the clearest distinction between the two profiles lay in overall activity level and screen-based sedentary behaviour.

**Table 4:** Behavioural Indicators across profiles

Variable	Lower-activity profile(*n* = 908)	Higher-activity profile(*n* = 148)	t	*p*	Cohen’s d
Sleep	541.27(60.04)	548.52(55.24)	-1.376	0.169	0.122
Learning	308.18(119.03)	334.86(111.38)	-2.550	0.011	0.226
Screen	241.17(103.16)	191.42(87.15)	6.266 ^a^	< 0.001	0.492
Other	87.48(31.49)	86.13(29.44)	0.487	0.627	0.043
VPA	30.96(26.72)	86.83(50.45)	-13.175 ^a^	< 0.001	1.795
MPA	27.63(17.76)	98.69(29.64)	-28.343 ^a^	< 0.001	3.579
LPA	76.26(63.44)	126.79(56.61)	-9.115	< 0.001	0.808

*M (SD)* mean (standard deviation). Between-profile differences were examined using independent-samples t tests. For variables violating the assumption of homogeneity of variance, Welch’s t test was used; these results are indicated by superscript a. Cohen’s *d* was calculated to indicate effect size

### Logistic regression analysis of demographic predictors

To examine the predictive effects of demographic variables on students’ membership in the different behavioural profiles, logistic regression was conducted using the R3STEP (robust three-step) approach, with the lower-activity profile serving as the reference group. The results are presented in Table 5. Gender showed a significant effect, with female students exhibiting a lower likelihood of belonging to the higher-activity profile than male students (OR = 0.446, 95% CI [0.294, 0.677], *p* < 0.001). In addition, junior students were more likely than first-year students to be classified into the higher-activity profile (OR = 1.979, 95% CI [1.044, 3.754], *p* = 0.037). Other demographic variables—including grade, residential area, only-child status, and family economic status—were not significant predictors of profile membership (*ps* > 0.05).

**Table 5:** Demographic predictors of profile membership

Variable	Higher-activity profile				
	B	SE	Z	*p*	OR[95% CI]
Gender (*Ref*. = Male)					
Female	-0.807	0.212	-3.800	< 0.001	0.446[0.294,0.677]
Grade (*Ref*. = Freshman)					
Sophomore	-0.155	0.254	-0.610	0.542	0.857[0.521,1.408]
Junior	0.683	0.327	2.091	0.037	1.979[1.044,3.754]
Senior	-0.022	0.427	-0.051	0.959	0.978[0.424,2.258]
Graduate Student	-0.089	0.416	-0.214	0.830	0.915[0.405,2.067]
Residential area (*Ref*. = Urban)					
Rural	0.083	0.222	0.375	0.708	1.087[0.703,1.679]
Only Child (*Ref*. = Yes)					
No	-0.018	0.235	-0.076	0.939	0.982[0.620,1.556]
Family Economic Status (*Ref*. = Relatively Poor)					
Average	0.053	0.254	0.208	0.835	1.054[0.641,1.735]
Relatively Wealthy	0.732	0.442	1.653	0.098	2.078[0.873,4.947]

*Ref*. Reference category, *B* logit estimation *SE* standard errors, *OR* odds ratio, *CI * confidence interval

### Differences in distal outcomes across 24-hour movement behaviour profiles

After examining demographic predictors of latent profile membership using the R3STEP approach, we further explored the associations between behavioural profiles and negative affect among university students. To obtain unbiased estimates of distal means while holding the latent classification invariant, the BCH method was employed to test the effects of profile membership on the distal outcomes. As shown in Table 6, students in the lower-activity profile reported significantly higher levels of depression, anxiety, and stress than those in the higher-activity profile. Specifically, significant between-profile differences were observed for depression (χ² = 10.493, *p* = 0.001), anxiety (χ² = 14.547, *p* < 0.001), and stress (χ² = 13.229, *p* < 0.001). Overall, these findings indicate that membership in the lower-activity profile was associated with less favourable emotional outcomes, whereas the higher-activity profile was associated with lower levels of negative affect.

**Table 6:** Negative affect scores across profiles

Variable	1 Lower-activity profile	2 Higher-activity profile	χ²(df)	*p*	Cohen’s d
Depression	6.621(0.276)	4.485(0.591)	10.493(1)	0.001	0.244
Anxiety	6.707(0.252)	4.565(0.497)	14.547(1)	< 0.001	0.273
Stress	7.797(0.276)	5.636(0.520)	13.229(1)	< 0.001	0.256

Values are presented as estimated means (standard errors). χ², chi-square. Because BCH output does not directly provide effect sizes, Cohen’s *d* values were additionally calculated based on the observed means and standard deviations by most likely class membership

## Discussion

Using latent profile analysis, the present study examined 24-hour movement behaviour patterns among Chinese university students and systematically investigated their demographic predictors and associations with negative affect. The original a priori hypotheses were only partially supported. Although we initially expected to identify approximately three to four profiles, the final retained main model was a more parsimonious two-profile solution, comprising a lower-activity profile and a higher-activity profile. Importantly, this two-profile solution was not specified a priori, but emerged from exploratory model comparison and was retained because it provided the clearest and most interpretable distinction. Integrating results from both the R3STEP and BCH procedures, the study demonstrates meaningful heterogeneity in students’ daily time allocation and highlights pronounced differences in negative emotional outcomes across the retained behavioural profiles.

### Characteristics of 24-hour movement behaviour profiles among university students

Consistent with previous research on 24-hour movement behaviours, this study identified two representative latent profiles among Chinese university students. The majority of students (86%) were classified into the lower-activity profile, characterised by lower levels of vigorous-, moderate-, and light-intensity physical activity and higher screen-based sedentary behaviour. In contrast, the higher-activity profile (14%) was marked by substantially greater engagement in physical activity across all three intensity levels, together with lower screen-based sedentary time. Although the higher-activity profile also showed slightly higher study-related sedentary behaviour, the most pronounced between-profile differences were observed in physical activity and screen-based sedentary behaviour. This pattern suggests that the clearest behavioural distinction among Chinese university students may not lie in sleep or overall sedentary time per se, but rather in the extent to which students maintain active lifestyles while limiting screen-based sedentary behaviour [10, 22]. A growing evidence base suggests that health-related behaviours tend to cluster in coordinated combinations. Within university settings, sedentary behaviour has become the predominant behavioural mode, while high-intensity physical activity represents a relatively small proportion of students [32]. Latent profile studies have further shown that high activity levels typically co-occur with lower sedentary time and healthier sleep patterns, whereas unfavourable behavioural combinations cluster in the opposite direction [21]. In this context, the predominance of the lower-activity profile in the present study may reflect a common behavioural configuration shaped by academic demands and digitally mediated lifestyles in Chinese higher education. By contrast, the higher-activity profile appears to represent a more adaptive minority who sustain physically active lifestyles despite similar academic pressures. Overall, the two-profile solution retained in the present study captures the most robust behavioural distinction in this sample, namely the contrast between a broadly lower-activity pattern and a more active pattern.

It is important to note, however, that this two-profile structure was not the originally hypothesised profile solution. The originally expected, more differentiated three-profile solution is reported in the Supplementary Materials. The three-profile solution provided some support for the anticipated sedentary-dominant, lightly active and high-activity structure, but the additional lightly active profile did not provide sufficiently distinct emotional-outcome information to warrant its use as the main model. Thus, the original expectation of a more differentiated profile structure and a clear gradient of negative affect across multiple profiles was not fully supported. Instead, the most robust distinction in both behavioural indicators and emotional outcomes was between students with lower overall activity and higher screen-based sedentary behaviour and those with higher activity levels and lower screen-based sedentary behaviour. For this reason, the two-profile solution was retained as a non-a-priori, exploratory main model.

### Demographic characteristics of latent profile membership

Using the R3STEP three-step approach, this study examined the predictive effects of demographic variables on profile membership while accounting for classification uncertainty. The results indicated that gender and academic year were significant predictors. Compared with male students, female students were less likely to belong to the higher-activity profile. This finding is consistent with extensive global evidence showing that women generally engage in lower levels of physical activity and are less likely to meet activity guidelines [33, 34]. Such patterns may be shaped by multiple factors, including gender socialisation, body image concerns, exercise self-efficacy and perceived safety. Furthermore, third-year students were more likely than first-year students to belong to the higher-activity profile. One possible explanation is that, having gradually adapted to university life, third-year students may have developed more stable self-regulatory strategies and healthier behavioural routines. Alternatively, the increased academic demands and pressures related to further education or employment in later years may prompt some students to engage in physical activity as a deliberate strategy to manage stress. By contrast, traditional sociodemographic indicators such as place of origin, only-child status and family economic background did not significantly predict profile membership. This suggests that, within this sample, variations in 24-hour movement behaviour patterns are not primarily driven by social stratification factors but may instead reflect a combination of individual preferences, campus environments and academic contexts.

### Associations between 24-hour movement behaviour profiles and negative affect

A key contribution of this study lies in its application of the BCH method to link latent profile with continuous emotional outcomes such as depression, anxiety and stress. Unlike traditional “classify–then–analyse” approaches, the BCH procedure corrects for classification error when estimating distal means, and is therefore widely recommended for examining associations between latent classes and continuous outcomes. The BCH results revealed a clear pattern in negative affect across behavioural profiles: students in the lower-activity profile reported higher levels of depression, anxiety and stress, whereas those in the higher-activity profile reported lower levels of these negative emotional outcomes. This pattern aligns closely with recent evidence on the combined effects of 24-hour movement behaviours on mental health. Systematic reviews and compositional data analyses have shown that a daily time-use composition characterised by higher moderate-to-vigorous physical activity, lower sedentary time and adequate sleep is strongly associated with reduced depression, anxiety and psychological distress. Research focusing on university students similarly indicates that lifestyle profiles marked by high sedentary time and low physical activity are linked to poorer mental health outcomes, whereas more favourable lifestyle clusters are associated with fewer depressive and anxiety symptoms [10, 35]. Within the Chinese university context, this study extends these findings by demonstrating consistent patterns under a 24-hour behavioural framework. The protective effects of the higher-activity profile may operate through multiple mechanisms. Physiologically, regular engagement in moderate-to-vigorous physical activity promotes the release of brain-derived neurotrophic factor, regulates hypothalamic–pituitary–adrenal axis functioning and reduces systemic inflammation, thereby strengthening the neurobiological foundations of emotion regulation [17]. Psychologically and behaviourally, active participation in physical exercise is associated with greater self-efficacy, improved interpersonal competence, stronger stress-coping capacity and richer social interaction, all of which represent important psychological resources for mitigating negative affect [36–38]. In contrast, students in the lower-activity profile may be exposed to a less favourable behavioural configuration, characterised by lower overall movement and greater screen-based sedentary time. Such a pattern may exacerbate emotional distress by increasing upward social comparison, disrupting daily routines and displacing time for restorative activities and face-to-face social interactions [39–41]. Importantly, the present findings suggest that the distinction between profiles was driven more strongly by physical activity and screen-based sedentary behaviour than by sleep. This does not imply that sleep is unimportant for mental health; rather, within the present sample, sleep played a less prominent role in differentiating behavioural subgroups than activity-related behaviours. Overall, these findings underscore the importance of an integrated 24-hour movement perspective and suggest that increasing physical activity while reducing screen-based sedentary behaviour may be especially beneficial for university students’ emotional wellbeing.

### Limitations and future directions

This study has several limitations. Its cross-sectional design prevents causal inference, and reverse or bidirectional relationships cannot be ruled out; longitudinal and latent transition approaches are needed to clarify temporal and causal pathways [42, 43]. All behavioural indicators were self-reported, which may introduce recall and social desirability bias, suggesting that future studies should incorporate accelerometers or other wearable devices to obtain more objective 24-hour movement data [44, 45]. In addition, important variables such as sleep quality, academic workload, social support and the campus activity environment were not examined, limiting deeper mechanistic interpretation [46]. Although the two-profile solution was retained as the main model for reasons of parsimony and interpretability, it was not the originally hypothesised profile structure. The original a priori hypothesis anticipated a more differentiated structure of approximately three to four profiles. The three-profile solution, which was more closely aligned with the original expectation, provides a more nuanced understanding of subgroup variation and may help inform future research. Finally, although participants were drawn from multiple regions, the sample may not represent all types of Chinese higher education institutions, and future research should replicate these findings using more diverse samples and cross-cultural comparisons to assess generalisability [47, 48].

## Conclusion

This study identified two 24-hour movement behaviour profiles among Chinese university students—a lower-activity profile and a higher-activity profile—with clear differences in depression, anxiety and stress. Students in the lower-activity profile reported less favourable emotional outcomes, whereas those in the higher-activity profile showed lower levels of negative affect. Demographic factors showed limited predictive value overall, although gender and academic year significantly predicted profile membership. These findings underscore the importance of an integrated 24-hour movement approach in mental health promotion, suggesting that reducing screen-based sedentary behaviour and increasing physical activity may support healthier behavioural profiles and improve university students’ emotional wellbeing. 

## Supplementary Information

Supplementary Material 1.

## Data Availability

The datasets generated and analysed during the current study are not publicly available due to concerns regarding participant confidentiality but are available from the corresponding author on reasonable request.

## Footnotes

1 ***Distal variables*** refer to continuous outcome variables that do not contribute to the formation of latent profile but are compared across profiles once the latent classification is established. In this study, the depression, anxiety and stress scores served as distal variables. The Bolck-Croon-Hagenaars (BCH) method was employed to estimate mean differences in these continuous distal outcomes across latent classes while correcting for classification error, thereby reducing bias associated with classification uncertainty.

## Abbreviations

24HMBQ: 24 Hour Movement Behaviour Questionnaire
DASS-21: Depression Anxiety Stress Scales-21
LPA: Latent Profile Analysis
R3STEP: Robust three-step
BCH: Bolck–Croon–Hagenaars
AIC: Akaike Information Criterion
BIC: Bayesian Information Criterion
LMR: Lo–Mendell–Rubin likelihood ratio test
BLRT: Bootstrap likelihood ratio test
SB: Sedentary behaviour
VPA: Vigorous physical activity
MPA: Moderate physical activity
