# Supplementary material for: Distinct 24-hour movement behaviour profiles and their associations with negative affect among Chinese university students
Source: BMC Psychol. 2026 May 29;14:1114. doi: 10.1186/s40359-026-04889-5 (PMC13410578; doi:10.1186/s40359-026-04889-5)
Supplement: Supplementary file 1 — Supplementary Material 1. [file 40359_2026_4889_MOESM1_ESM.docx]

Online Supplementary Information for –“*Distinct 24-hour movement behaviour profiles and their associations with negative affect among Chinese university students*”

**Contents**

**Section 1. Measurement Instruments**

- - Supplementary Table S1. Chinese versions of the 24HMBQ and DASS-21
  - Supplementary Table S2. English translations of the 24HMBQ and DASS-21

**Section 2. Psychometric evaluation of the DASS-21**

- - Supplementary Figure S1. Confirmatory Factor Analysis Model
  - Supplementary Table S3. CFA fit indices, composite reliability, and convergent validity
  - Supplementary Table S4. Discriminant validity of the negative affect scale

**Section 3. Supplementary three-profile solution corresponding to the original a priori expectation**

- - Supplementary Table S5. Fit indices for the supplementary three-profile solution
  - Supplementary Figure S2. Supplementary three-profile model of 24-hour movement behaviours
  - Supplementary Table S6. Demographic composition across profiles in the retained two-profile solution
  - Supplementary Table S7. Demographic composition across profiles in the supplementary three-profile solution
  - Supplementary Table S8. Multinomial logistic regression analysis within the supplementary three-profile solution
  - Supplementary Table S9. Additional comparison between the high-activity and lightly active profiles
  - Supplementary Table S10. Differences in negative affect across profiles in the supplementary three-profile solution

**Section 1**

**Measurement Instruments**

To enhance transparency and facilitate international understanding of the study instruments, the supplementary materials provide the original Chinese and English-translated versions of the Chinese College Student 24-Hour Movement Behaviour Questionnaire (24HMBQ) and the Depression Anxiety Stress Scales-21 (DASS-21). Supplementary Table S1 presents the original Chinese versions of the instruments, and Supplementary Table S2 presents the corresponding English translations. The 24HMBQ assesses sleep, sedentary behaviour, and physical activity, whereas the DASS-21 assesses depression, anxiety, and stress. By providing bilingual versions of these instruments, the present study aims to improve transparency and support cross-cultural interpretation and future replication.

Supplementary Table S1.

**中国大学生24小时活动行为问卷（24HMBQ）**(Zheng et al., 2023)

***睡眠情况***

请回忆您最近七天的睡眠情况，填写具体的时间（24小时制）和时长。

| 题目 | 工作日 | 周末 |
| --- | --- | --- |
| 最近一周内，您通常每天晚上入睡的时间是? | ： | ： |
| 最近一周内，您通常每天早上起床的时间是? | ： | ： |
| 最近一周内，您通常每天白天小憩的时长是?(注:小憩是一段短暂的睡眠时间，包括午休) | __小时 分钟 | __小时 分钟 |

***久坐行为***

请回忆您最近七天的久坐行为，填写具体的时长和频率。

| 题目 | 工作日 | 周末 |
| --- | --- | --- |
| 最近一周内，您平均每天坐着学习 (包括听课、自习等) 或工作的时长是? | __小时 分钟 | __小时 分钟 |
| 最近一周内，您每天在学习或工作的过程中，平均间隔多长时间离开座位一次? (例如:站起来放松活动、去茶水间等) | ○＜30分钟/次  ○≥30分钟且＜60分钟/次  ○≥60分钟且＜90分钟/次  ○≥90分钟且＜120分钟/次  ○≥120分钟 | |
| 最近一周内，您平均每天坐着或躺着使用电子屏幕设备进行休闲娱乐类活动的时长是? (注:此类活动主要是指除学习或工作需要之外使用观看电子屏幕设备，例如: 看电视/电影/短视频、玩电子游戏、使用社交媒体等。) | __小时 分钟 | __小时 分钟 |
| 最近一周内，您每天使用电子屏慕设备进行休闲娱乐类活动时，平均间隔多长时间离开座位一次? | ○＜30分钟/次  ○≥30分钟且＜60分钟/次  ○≥60分钟且＜90分钟/次  ○≥90分钟且＜120分钟/次  ○≥120分钟 | |

***身体活动***

| 请回忆您最近七天的身体活动情况，填写具体的时长和频率   \| 题目 \| 运动锻炼 (包括各类健身运动、上体育课等) \| 日常出行 \| 日常宿舍生活 \| \| --- \| --- \| --- \| --- \| \| 您在最近一周内进行高强度身体活动(感觉费力，呼吸急促，心率明显加快)的频率和时长是? \| 次/周;每次 小时___分钟 \| 次/周;每次 小时 分钟  (例如:快速跨车骑行等) \| 次/周;每次 小时 分钟  (例如: 搬重物等) \| \| 您在最近一周内进行中等强度身体活动(感觉有些费力，呼吸加快，心率明显加快)的频率和时长是? \| 次/周;每次 小时___分钟 \| 次/周;每次 小时 分钟  (例如:快速步行、滑板出行、慢速蹬车骑行等) \| 次/周;每次 小时 分钟  (例如:搬轻物，打扫卫生等） \| \| 您在最近一周内进行低强度身体活动(不太费力，呼吸和心率无明显加快)的频率是? \| 次/周;每次 小时___分钟 \| 次/周;每次 小时 分钟  (例如: 步行等) \| 次/周;每次 小时 分钟  (例如:整理内务、清洗衣物等) \| \| 最近一周内，您进行了几次涉及到主要肌群骨骼肌的力量练习? \| 次/周  (例如: 力量器械训练、俯卧撑等无器械力量练习等) \| \| \| |
| --- | --- | --- | --- | --- | --- | --- | --- | --- | --- | --- | --- | --- | --- | --- | --- | --- | --- | --- | --- | --- |

**抑郁-焦虑-压力量表（DASS-21）**(龚栩 et al., 2010)

请您根据过去一周的情况，在每个条目中选择适用于你情况的程度选择。请仔细阅读并回答每个条目，选择没有对错之分。（不符合-非常符合4点计分）

| 1.我觉得很难让自己安静下来。 |
| --- |
| 2.我感到口干舌燥。 |
| 3.我好像一点都没有感觉到任何愉快，舒畅。 |
| 4.我感到呼吸困难（例如:气喘或透不过气来）。 |
| 5.我感到很难主动去开始工作。 |
| 6.我对事情往往做出过敏反应。 |
| 7.我感到颤抖（例如:手抖）。 |
| 8.我觉得自己消耗了很多精力。 |
| 9.我担心一些可能让自己恐慌或者出丑的场合。 |
| 10.我觉得对自己不久的将来没有什么可期盼的。 |
| 11.我感到忐忑不安。 |
| 12.我感到很难放松自己。 |
| 13.我感到忧郁沮丧。 |
| 14.我无法容忍任何阻碍我继续工作的事情。 |
| 15.我感到快要崩溃了。 |
| 16.我对任何事情都不能产生热情。 |
| 17.我觉得自己不怎么配做人。 |
| 18.我发觉自己很容易被触怒。 |
| 19.即使在没有明显的体力活动时，我也感到心律不正常。 |
| 20.我无缘无故的感到害怕。 |
| 21.我感到生命毫无意义。 |

Supplementary Table S2.

***Chinese college student 24-Hour movement behavior questionnaire (24HMBQ)*** (Zheng et al., 2023)

***Sleep***

Please recall your sleep patterns over the past week, and fill in the specific times (24-hour format) and durations.

| Question | Weekdays | Weekend |
| --- | --- | --- |
| In the past week, what time did you usually go to bed at night? | ： | ： |
| In the past week, what time did you usually get up in the morning? | ： | ： |
| In the past week, what was the usual duration of your daytime naps? (Note: A nap is a short period of sleep, including a lunch break.) | __ hours  __ minutes | __ hours  __ minutes |

***Sedentary Behavior***

Please recall your sedentary behaviors over the past seven days and provide the specific durations and frequencies.

| Question | Weekdays | Weekend |
| --- | --- | --- |
| In the past week, what was your average daily time spent sitting while learning (including attending lectures, self-study, etc.) or working? | __ hours  __ minutes | __ hours  __ minutes |
| During learning or work in the past week, on average, how often did you get up from your seat? (e.g., to stand up and stretch, go to the pantry, etc.) | ○ < 30 minutes/time ○ ≥ 30 minutes & < 60 minutes/time ○ ≥ 60 minutes & < 90 minutes/time ○ ≥ 90 minutes & < 120 minutes/time ○ ≥ 120 minutes | |
| In the past week, what was your average daily time spent sitting or lying down using electronic screen devices for leisure entertainment? (Note: This primarily refers to using electronic screens for purposes other than learning or work needs, e.g., watching TV/movies/short videos, playing video games, using social media, etc.) | __ hours  __ minutes | __ hours  __ minutes |
| While using electronic screen devices for leisure entertainment in the past week, on average, how often did you get up from your seat? | ○ < 30 minutes/time ○ ≥ 30 minutes & < 60 minutes/time ○ ≥ 60 minutes & < 90 minutes/time ○ ≥ 90 minutes & < 120 minutes/time ○ ≥ 120 minutes | |

***Physical Activity***

| Please recall your physical activity over the past seven days and provide the specific frequency and duration.   \| Question \| Exercise  (Includes various fitness sports, physical education classes, etc.) \| Active Transportation \| Daily Dormitory Life \| \| --- \| --- \| --- \| --- \| \| In the past week, what was the frequency and duration of your **Vigorous-intensity physical activity**? (Feels exhausting, breathing is rapid, heart rate increases substantially) \| __ times/week;  Each time __ hours __ minutes \| __ times/week; Each time __ hours __ minutes (e.g., fast bicycling, etc.) \| __times/week; Each time __ hours __ minutes (e.g., moving heavy objects, etc.) \| \| In the past week, what was the frequency and duration of your **Moderate-intensity physical activity**? (Feels somewhat exhausting, breathing accelerates, heart rate increases noticeably) \| ____ times/week; Each time __ hours __ minutes \| __ times/week; Each time __ hours __ minutes (e.g., brisk walking, skateboarding, slow bicycling, etc.) \| __times/week; Each time __ hours __ minutes (e.g., moving light objects, cleaning, etc.) \| \| In the past week, what was the frequency and duration of your **Light-intensity physical activity**? (Not very exhausting, no noticeable increase in breathing or heart rate) \| __ times/week; Each time __ hours __ minutes \| __ times/week; Each time __ hours __ minutes (e.g., walking, etc.) \| __times/week; Each time __ hours __ minutes (e.g., tidying up the room, washing clothes, etc.) \| \| In the past week, how many times did you perform muscle-strengthening activities involving major muscle groups? \| __ times/week (e.g., strength machine training, bodyweight exercises like push-ups, etc.) \| \| \| |
| --- | --- | --- | --- | --- | --- | --- | --- | --- | --- | --- | --- | --- | --- | --- | --- | --- | --- | --- | --- | --- |

***Depression anxiety stress scales (DASS-21)*** (Gong et al., 2010)

Please read each statement and select the option that best applies to you **over the past week**. Read and answer each item carefully. There are no right or wrong answers. (4-point scale: Did not apply to me at all - Applied to me very much, or most of the time)

| 1.I find it hard to calm down. |
| --- |
| 2.I feel dry in the mouth. |
| 3.I feel like I cannot experience any joy or comfort. |
| 4.I feel short of breath (e.g., out of breath or can’t catch my breath). |
| 5.I find it hard to start work. |
| 6.I often react excessively to things. |
| 7.I feel trembling (e.g., hands shaking). |
| 8.I feel like I have used up a lot of energy. |
| 9.I worry about situations that may cause panic or embarrassment. |
| 10.I feel there is nothing to look forward to in the near future. |
| 11.I feel uneasy. |
| 12.I find it hard to relax. |
| 13.I feel depressed or downhearted. |
| 14.I cannot tolerate anything that interrupts my work. |
| 15.I feel like I’m about to break down. |
| 16.I cannot get excited about anything. |
| 17.I feel unworthy of being a person. |
| 18.I find myself getting easily irritated. |
| 19.Even without physical activity, I feel like my heart rate is irregular. |
| 20.I feel fear for no reason. |
| 21.I feel life is meaningless. |

**References**

Zheng, J., Tan, T. C., Zheng, K., & Huang, T. (2023). Development of a 24-hour movement behaviors questionnaire (24HMBQ) for Chinese college students: Validity and reliability testing. BMC Public Health, 23(1), 752.

<https://doi.org/10.1186/s12889-023-15393-5>

Gong, X., Xie, X., Xu, R., & Luo, Y. (2010). Psychometric properties of the Chinese version of the Depression Anxiety Stress Scale–21 (DASS-21) in Chinese college students. Chinese Journal of Clinical Psychology, 18(4), 443–446.

<https://doi.org/10.16128/j.cnki.1005-3611.2010.04.020>

**Section 2**

**Psychometric evaluation of the DASS-21**


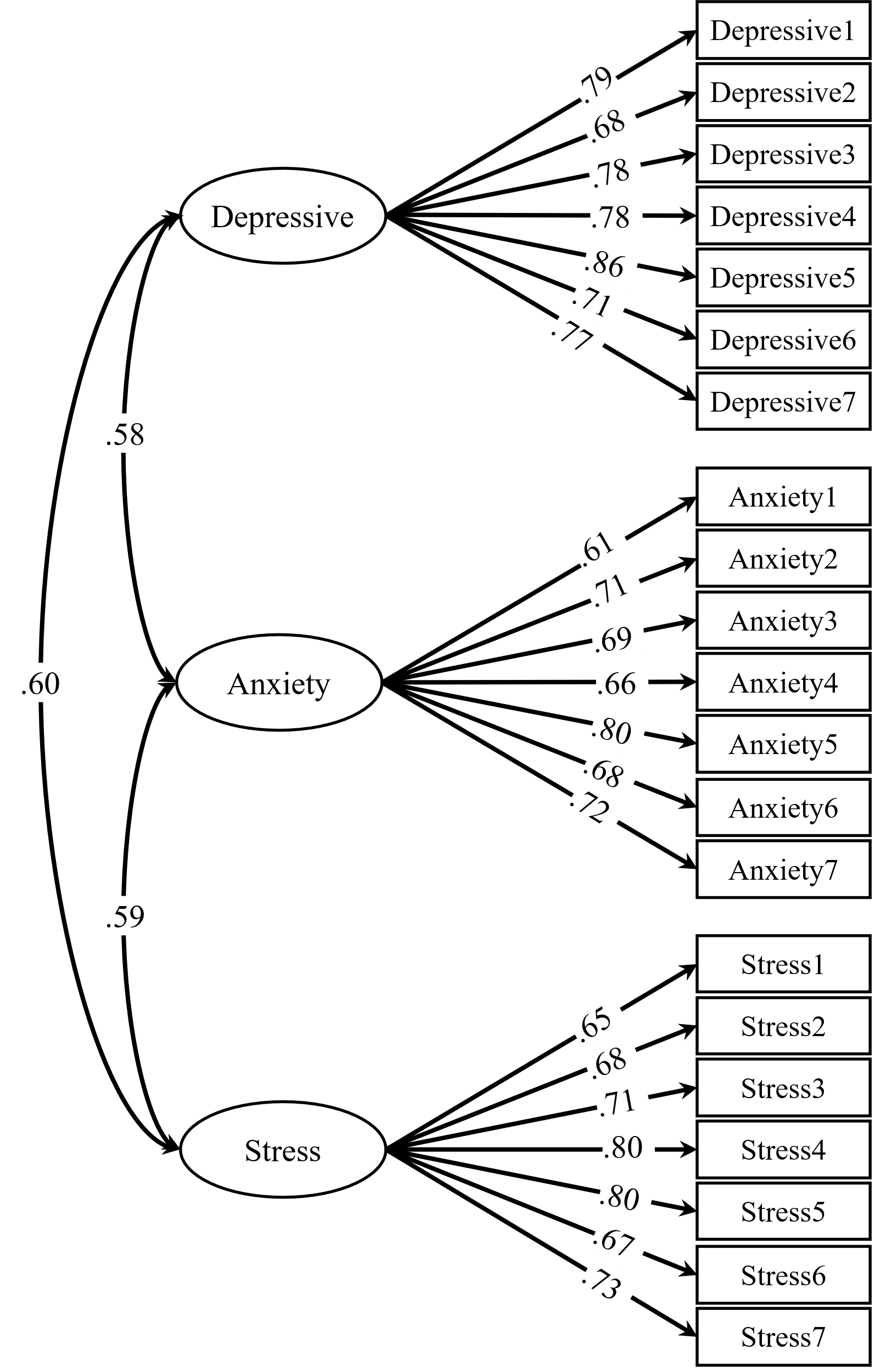


**Supplementary Figure S1. Confirmatory Factor Analysis Model**

To evaluate the psychometric properties of the DASS-21 in the present sample, a confirmatory factor analysis (CFA) was conducted using AMOS 27.0 (Supplementary Figure S1). As shown in Supplementary Table S3, the three-factor model demonstrated good model fit: χ²(186) = 1022.923, CFI = 0.929, TLI = 0.920, RMSEA = 0.065, and SRMR = 0.049, all of which met or exceeded recommended criteria (Hu & Bentler, 1999).

Regarding convergent validity, all standardized factor loadings ranged from 0.614 to 0.855, and composite reliability (CR) values ranged from 0.868 to 0.908, indicating good internal consistency. Although the average variance extracted (AVE) for the Anxiety dimension was slightly below the 0.50 threshold (AVE = 0.486), its high CR value and significant loadings suggest acceptable convergent validity (Fornell & Larcker, 1981). For discriminant validity (Supplementary Table S4), the square roots of the AVEs exceeded the inter-factor correlations, and the additional HTMT (Heterotrait-Monotrait Ratio) indices (Henseler et al., 2015) were all below 0.85, further supporting adequate discriminant validity among the three factors.

Overall, the results indicate that the DASS-21 exhibits satisfactory measurement quality in this study and is suitable for subsequent analyses.

| Supplementary Table S3 | | | | |
| --- | --- | --- | --- | --- |
| CFA Fit Indices, Composite Reliability (CR), and Convergent Validity (AVE). | | | | |
| Negative Affect | Items | Standardized Factor Loadings | CR | AVE |
| Depressive | Depressive1 | 0.788 | 0.908 | 0.588 |
|  | Depressive2 | 0.676 |  |  |
|  | Depressive3 | 0.784 |  |  |
|  | Depressive4 | 0.777 |  |  |
|  | Depressive5 | 0.855 |  |  |
|  | Depressive6 | 0.705 |  |  |
|  | Depressive7 | 0.767 |  |  |
| Anxiety | Anxiety1 | 0.614 | 0.868 | 0.486 |
|  | Anxiety2 | 0.708 |  |  |
|  | Anxiety3 | 0.692 |  |  |
|  | Anxiety4 | 0.661 |  |  |
|  | Anxiety5 | 0.797 |  |  |
|  | Anxiety6 | 0.677 |  |  |
|  | Anxiety7 | 0.716 |  |  |
| Stress | Stress1 | 0.649 | 0.883 | 0.521 |
|  | Stress2 | 0.677 |  |  |
|  | Stress3 | 0.710 |  |  |
|  | Stress4 | 0.803 |  |  |
|  | Stress5 | 0.798 |  |  |
|  | Stress6 | 0.670 |  |  |
|  | Stress7 | 0.728 |  |  |
| Model Fit | | | | |
| χ^2^ | 1022.923 | | | |
| *df* | 186 | | | |
| CFI | 0.929 | | | |
| TLI | 0.920 | | | |
| RMSEA | 0.065 | | | |
| SRMR | 0.049 | | | |

| Supplementary Table S4 | | | |
| --- | --- | --- | --- |
| Discriminant Validity of the Negative Affect Scale. | | | |
| Factor | Depressive | Anxiety | Stress |
| Depressive | **0.767** | 0.572 | 0.585 |
| Anxiety | 0.576 | **0.697** | 0.606 |
| Stress | 0.589 | 0.604 | **0.722** |
| ***Note****:* Diagonal values in bold represent the square roots of the AVEs. The lower triangle shows factor correlations, and the upper triangle shows HTMT values. | | | |

**References**

Hu, L. T., & Bentler, P. M. (1999). Cutoff criteria for fit indexes in covariance structure analysis: Conventional criteria versus new alternatives. Structural Equation Modeling, 6(1), 1-55.

<https://doi.org/10.1080/10705519909540118>

Fornell, C., & Larcker, D. F. (1981). Structural Equation Models with Unobservable Variables and Measurement Error. Journal of Marketing Research, 18(1), 39–50.

<https://doi.org/10.2307/3151312>

Henseler J., Ringle C. M., SARSTEDT M. A new criterion for assessing discriminant validity in variance-based structural equation modeling[J]. Journal of the Academy of Marketing Science, 2015,43(1): 115-135.

**Section 3**

**Supplementary three-profile solution corresponding to the original a priori expectation**

Because the original a priori hypothesis anticipated approximately three to four profiles, the three-profile solution was further examined as a supplementary model. Although this solution was more closely aligned with the originally expected profile structure, the two-profile solution was retained as the main model in the manuscript because it provided a more parsimonious and interpretable distinction between lower-activity and higher-activity behavioural patterns. Accordingly, the results reported in this section are presented as supplementary evidence rather than as the primary analytical model.

| Supplementary Table S5  Fit indices for the supplementary three-profile solution. | | | | | | | |
| --- | --- | --- | --- | --- | --- | --- | --- |
| Model | AIC | BIC | aBIC | Entropy | LMR | BLRT | Group size for each profile(%) |
| 1-profile | 80469.59 | 80539.06 | 80494.60 | - | - | - | - |
| 2-profile | 79830.42 | 79939.59 | 79869.71 | 0.911 | *<* 0.001 | *<* 0.001 | 0.86/0.14 |
| **3-profile** | **79594.49** | **79743.36** | **79648.08** | **0.865** | **0.047** | ***<* 0.001** | **0.13/0.73/0.14** |
| 4-profile | 79427.17 | 79615.74 | 79495.04 | 0.879 | 0.386 | *<* 0.001 | 0.71/0.13/0.14/0.02 |
| 5-profile | 79215.72 | 79443.99 | 79297.88 | 0.882 | 0.008 | *<* 0.001 | 0.12/0.07/0.11/0.68/0.02 |
| ***Note***: Values in bold denote the selected model. AIC = Akaike Information Criterion; BIC = Bayesian Information Criterion; aBIC = adjusted BIC; LMR = *p* values for Lo-Mendell-Rubin adjusted likelihood ratio test for K vs. K-1 profiles; BLRT = *p* values for Bootstrapped Likelihood Ratio Test. | | | | | | | |

As shown in Supplementary Table S5, the three-profile solution demonstrated acceptable statistical fit and yielded three descriptively distinguishable subgroups. However, compared with the retained two-profile solution in the main text, the three-profile model was less parsimonious and added interpretive complexity. It was therefore reported as a supplementary model corresponding to the original a priori expectation rather than retained as the primary solution.


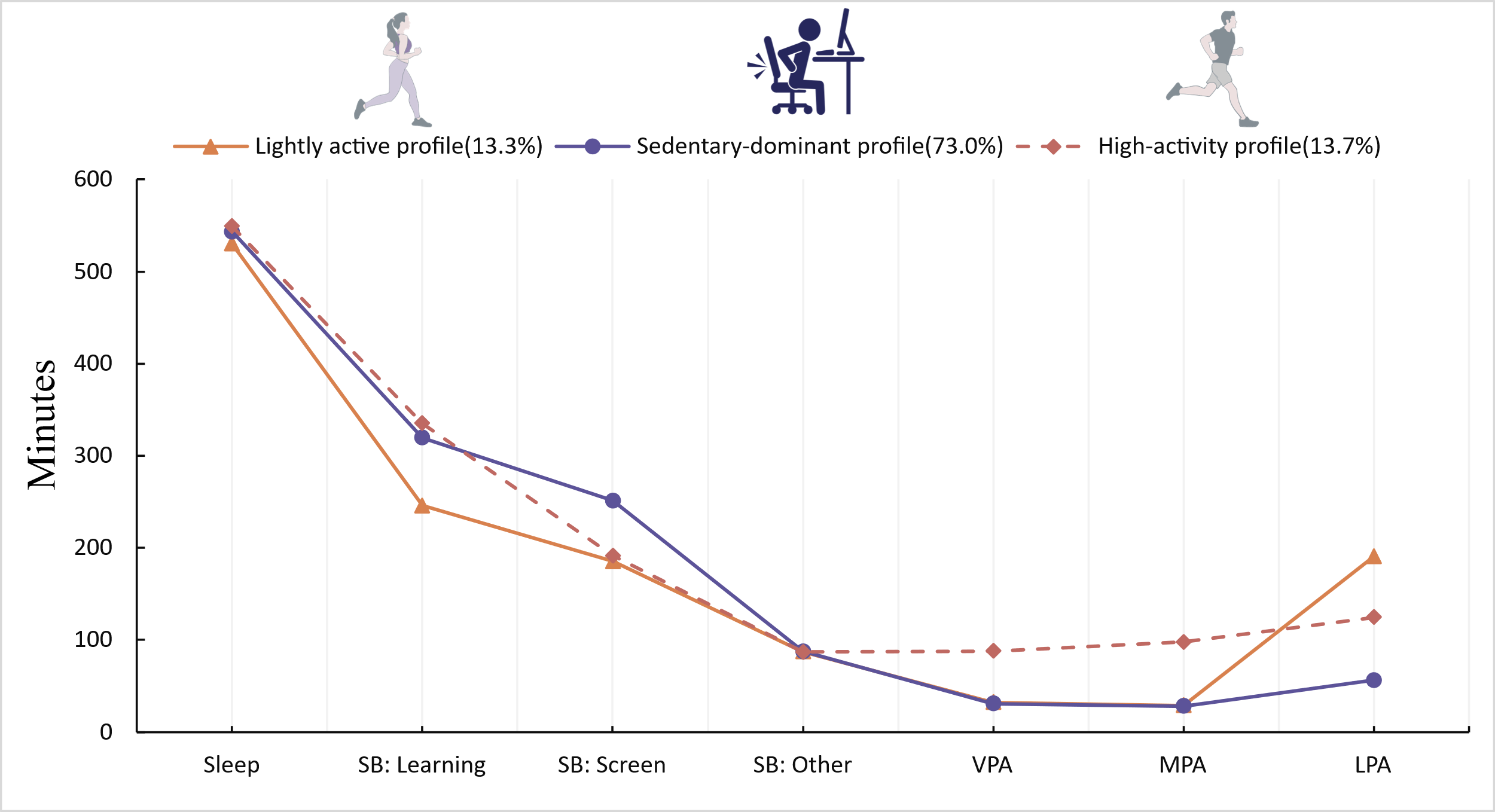


**Supplementary Figure S2. Supplementary three-profile model of 24-hour movement behaviours**

***Note: SB, sedentary behaviour; VPA, vigorous physical activity; MPA, moderate physical activity; LPA, light physical activity. The three profiles were labelled as the lightly active profile, the sedentary-dominant profile, and the high-activity profile. This model corresponds more closely to the original a priori expectation and is presented as supplementary evidence, whereas the two-profile solution was retained as the primary solution in the main text.***

| Supplementary Table S6 | | | |
| --- | --- | --- | --- |
| Demographic composition across profiles in the retained two-profile solution. | | | |
| Variable | Category | Lower-activity profile *n*(%) | Higher-activity profile *n*(%) |
| Gender | Male | 352(80.7%) | 84(19.3%) |
|  | Female | 556(89.7%) | 64(10.3%) |
| Grade | Freshman | 454(85.7%) | 76(14.3%) |
|  | Sophomore | 267(88.7%) | 34(11.3%) |
|  | Junior | 55(76.4%) | 17(23.6%) |
|  | Senior | 68(86.1%) | 11(13.9%) |
|  | Graduate Student | 64(86.5%) | 10(13.5%) |
| Residential area | Urban | 340(86.1%) | 55(13.9%) |
|  | Rural | 568(85.9%) | 93(14.1%) |
| Only child | Yes | 244(84.7%) | 44(15.3%) |
|  | No | 664(86.5%) | 104(13.5%) |
| Family economic status | Relatively poor | 190(86.0%) | 31(14.0%) |
|  | Average | 671(86.6%) | 104(13.4%) |
|  | Relatively wealthy | 47(78.3%) | 13(21.7%) |

| Supplementary Table S7 | | | | |
| --- | --- | --- | --- | --- |
| Demographic composition across profiles in the supplementary three-profile solution. | | | | |
| Variable | Category | Lightly active *n*(%) | Sedentary-dominant *n*(%) | High-activity *n*(%) |
| Gender | Male | 69(15.8%) | 285(65.4%) | 82(18.8%) |
|  | Female | 71(11.5%) | 486(78.4%) | 63(10.2%) |
| Grade | Freshman | 72(13.6%) | 382(72.1%) | 76(14.3%) |
|  | Sophomore | 42(14.0%) | 227(75.4%) | 32(10.6%) |
|  | Junior | 6(8.3%) | 48(66.7%) | 18(25.0%) |
|  | Senior | 12(15.2%) | 58(73.4%) | 9(11.4%) |
|  | Graduate Student | 8(10.8%) | 56(75.7%) | 10(13.5%) |
| Residential area | Urban | 55(13.9%) | 288(72.9%) | 52(13.2%) |
|  | Rural | 85(12.9%) | 483(73.1%) | 93(14.1%) |
| Only child | Yes | 38(13.2%) | 207(71.9%) | 43(14.9%) |
|  | No | 102(13.3%) | 564(73.4%) | 102(13.3%) |
| Family economic status | Relatively poor | 37(16.7%) | 152(68.8%) | 32(14.5%) |
|  | Average | 97(12.5%) | 576(74.3%) | 102(13.2%) |
|  | Relatively wealthy | 6(10.0%) | 43(71.7%) | 11(18.3%) |

***Note: Percentages are calculated within each demographic subgroup. Supplementary Table S6 corresponds to the retained primary two-profile solution, whereas Supplementary Table S7 corresponds to the supplementary three-profile solution.***

Supplementary Tables S6 and S7 present the demographic composition of the retained two-profile solution and the supplementary three-profile solution, respectively. In the retained two-profile solution, the lower-activity profile accounted for the majority of students across all demographic subgroups, whereas the higher-activity profile represented a smaller proportion. In the supplementary three-profile solution, the sedentary-dominant profile comprised the largest proportion across nearly all demographic categories, whereas the lightly active and high-activity profiles were smaller. Descriptively, male students and junior students appeared more likely to be classified into the more active subgroup(s) in both solutions. These patterns provide additional descriptive insight into subgroup variation, but the three-profile solution should be interpreted as supplementary evidence because it was not retained as the primary analytical model.

| Supplementary Table S8 | | | | | | | | |
| --- | --- | --- | --- | --- | --- | --- | --- | --- |
| Multinomial logistic regression analysis within the supplementary three-profile solution. | | | | | | | | |
| Variable | Lightly active profile | | | | High-activity profile | | | |
|  | B | SE | *Z* | OR[95% CI] | B | SE | *Z* | OR[95% CI] |
| Gender (*Ref*. = Male) | | | | | | | | |
| Female | -0.589 | 0.23 | -2.565^*^ | 0.56[0.354,0.87] | -0.853 | 0.217 | -3.931^***^ | 0.43[0.279,0.652] |
| Grade (*Ref*. = Freshman) | | | | | | | | |
| Sophomore | 0.056 | 0.262 | 0.215 | 1.06[0.633,1.769] | -0.231 | 0.263 | -0.88 | 0.79[0.474,1.328] |
| Junior | -0.421 | 0.584 | -0.72 | 0.66[0.209,2.063] | 0.701 | 0.325 | 2.154^*^ | 2.02[1.065,3.815] |
| Senior | 0.306 | 0.418 | 0.731 | 1.36[0.598,3.084] | -0.223 | 0.468 | -0.475 | 0.80[0.320,2.005] |
| Graduate Student | -0.287 | 0.497 | -0.578 | 0.75[0.284,1.986] | -0.121 | 0.415 | -0.291 | 0.89[0.393,1.998] |
| Residential area (*Ref*. = Urban) | | | | | | | | |
| Rural | -0.18 | 0.24 | -0.751 | 0.84[0.522,1.337] | 0.108 | 0.225 | 0.481 | 1.11[0.717,1.732] |
| Only Child (*Ref*. = Yes) | | | | | | | | |
| No | 0.068 | 0.26 | 0.26 | 1.07[0.642,1.783] | -0.033 | 0.238 | -0.137 | 0.97[0.607,1.543] |
| Family Economic Status (*Ref*. = Relatively Poor) | | | | | | | | |
| Average | -0.392 | 0.276 | -1.419 | 0.68[0.393,1.161] | -0.082 | 0.255 | -0.32 | 0.92[0.559,1.519] |
| Relatively Wealthy | -0.704 | 0.668 | -1.055 | 0.49[0.134,1.830] | 0.383 | 0.455 | 0.84 | 1.47[0.601,3.578] |
| ***Note***: *Ref*. = Sedentary-dominant profile; B, logit estimation; SE, standard errors; OR, odds ratio; ^*^*p <* 0.05; ^***^*p <* 0.001. | | | | | | | | |

Supplementary Table S8 presents the multinomial logistic regression results for the supplementary three-profile solution, with the sedentary-dominant profile as the reference group. Compared with male students, female students were less likely to be classified into either the lightly active profile or the high-activity profile rather than the sedentary-dominant profile. In addition, junior students were more likely than freshmen to belong to the high-activity profile. No other demographic variable showed a significant association with profile membership in this supplementary model. Overall, these results suggest that gender and grade may contribute to differences in subgroup membership, but the findings should be interpreted cautiously because the three-profile solution was reported as supplementary evidence rather than as the primary analytical model.

| Supplementary Table S9 | | | | |
| --- | --- | --- | --- | --- |
| Additional multinomial logistic regression analysis directly comparing the lightly active and high-activity profiles. | | | | |
| Variable | High-activity profile | | | |
|  | B | SE | *Z* | OR[95% CI] |
| Gender (*Ref*. = Male) | | | | |
| Female | -0.264 | 0.292 | -0.904 | 0.77[0.433,1.361] |
| Grade (*Ref*. = Freshman) | | | | |
| Sophomore | -0.288 | 0.342 | -0.841 | 0.75[0.384,1.466] |
| Junior | 1.122 | 0.628 | 1.786 | 3.07[0.896,10.51] |
| Senior | -0.529 | 0.578 | -0.914 | 0.59[0.190,1.830] |
| Graduate Student | 0.166 | 0.609 | 0.272 | 1.18 [0.358,3.898] |
| Residential area (*Ref*. = Urban) | | | | |
| Rural | 0.289 | 0.3 | 0.961 | 1.34[0.741,2.404] |
| Only Child (*Ref*. = Yes) | | | | |
| No | -0.101 | 0.32 | -0.314 | 0.90[0.483,1.695] |
| Family Economic Status (*Ref*. = Relatively Poor) | | | | |
| Average | 0.31 | 0.343 | 0.906 | 1.36[0.697,2.670] |
| Relatively Wealthy | 1.087 | 0.748 | 1.453 | 2.97[0.684,12.85] |

***Note*:** The lightly active profile served as the reference profile. B, logit coefficient; SE, standard error; OR, odds ratio.

Supplementary Table S9 provides an additional comparison between the high-activity profile and the lightly active profile within the supplementary three-profile solution. None of the demographic variables significantly distinguished these two profiles. Although some variables, such as junior grade and relatively wealthy family economic status, showed descriptively higher odds of membership in the high-activity profile, the corresponding estimates were not statistically significant. These findings suggest that the two more active subgroups were not clearly differentiated by the demographic variables examined in the present study.

| Supplementary Table S10  Differences in negative affect across supplementary profiles. | | | | | | |
| --- | --- | --- | --- | --- | --- | --- |
| Variable | 1 Lightly active profile | 2 Sedentary-dominant profile | 3 High-activity profile | Comparison | χ² (*p*) | Cohen’s *d* |
| Depression | 5.763(0.823) | 6.709(0.308) | 4.776(0.630) | Overall | 7.673(0.022) |  |
|  |  |  |  | 1 vs. 2 | 1.066(0.302) | 0.094 |
|  |  |  |  | 1 vs. 3 | 0.893(0.345) | 0.127 |
|  |  |  |  | 2 vs. 3 | 7.407(0.006) | 0.218 |
| Anxiety | 5.487(0.784) | 6.915(0.281) | 4.568(0.502) | Overall | 16.960 (< 0.001) |  |
|  |  |  |  | 1 vs. 2 | 2.696(0.101) | 0.151 |
|  |  |  |  | 1 vs. 3 | 0.970(0.325) | 0.148 |
|  |  |  |  | 2 vs. 3 | 16.370 (< 0.001) | 0.298 |
| Stress | 6.749(0.816) | 7.983(0.309) | 5.586(0.531) | Overall | 15.241 (< 0.001) |  |
|  |  |  |  | 1 vs. 2 | 1.840(0.175) | 0.121 |
|  |  |  |  | 1 vs. 3 | 1.413(0.235) | 0.171 |
|  |  |  |  | 2 vs. 3 | 14.999 (< 0.001) | 0.282 |
| ***Note***: This section presents the mean values and standard errors for each indicator of negative emotion across the different profiles, χ², chi-square. Because BCH output does not directly provide effect sizes, Cohen’s *d* values were additionally calculated based on the observed means and standard deviations by most likely class membership. | | | | | | |

Supplementary Table S10 shows differences in depression, anxiety, and stress across the supplementary three-profile solution. Although the overall differences were statistically significant, post hoc comparisons indicated that these differences were primarily driven by the contrast between the sedentary-dominant profile and the high-activity profile. By contrast, the lightly active profile did not differ significantly from either of the other two profiles across any of the three negative affect indicators. This pattern suggests that, although the three-profile solution provided additional behavioural differentiation and was broadly consistent with the original a priori expectation, it did not produce three clearly distinct groups in terms of negative emotional outcomes. From the perspective of distal outcome interpretation, this finding further supports retaining the more parsimonious two-profile solution as the primary model.
